# Protocol for socioecological study of autism, suicide risk, and mental health care: Integrating machine learning and community consultation for suicide prevention

**DOI:** 10.1371/journal.pone.0319396

**Published:** 2025-03-19

**Authors:** Nicole M. Marlow, Jessica M. Kramer, Anne V. Kirby, Molly M. Jacobs

**Affiliations:** 1 Department of Health Services, Research, Management and Policy, College of Public Health and Health Professions, University of Florida, Gainesville, Florida, United States of America; 2 Department of Occupational Therapy, College of Public Health and Health Professions, University of Florida, Gainesville, Florida, United States of America; 3 Department of Occupational & Recreational Therapies, College of Health, University of Utah, Salt Lake City, Utah, United States of America; Public Library of Science, UNITED KINGDOM OF GREAT BRITAIN AND NORTHERN IRELAND

## Abstract

**Introduction:**

Autistic people experience higher risk of suicidal ideation (SI) and suicide attempts (SA) compared to non-autistic people, yet there is limited understanding of complex, multilevel factors that drive this disparity. Further, determinants of mental health service receipt among this population are unknown. This study will identify socioecological factors associated with increased risk of SI and SA for autistic people and evaluate determinants of mental health care receipt.

**Methods:**

This study will link information for individuals aged 12-64 years in healthcare claims data (IBM® MarketScan® Research Database and CMS Medicaid) to publicly available databases containing community and policy factors, thereby creating a unique, multilevel dataset that includes health, demographic, community, and policy information. Machine learning data reduction methods will be applied to reduce the dimensionality prior to nested, multilevel empirical estimation. These techniques will allow for robust identification of clusters of socioecological factors associated with 1) risk of SI and SA and 2) receipt of mental health services (type, dose, delivery modality). Throughout, the research team will partner with an established group of autistic partners to promote community relevance, as well as receive input and guidance from a council of policy and practice advisors.

**Discussion:**

We hypothesize that nested individual (co-occurring conditions, age, sex), community (healthcare availability, social vulnerabilities), and policy factors (state mental health legislation, state Medicaid expansion) will be associated with heightened risk of SI and SA, and that receipt, dose, and delivery of mental health services will be associated with interdependent factors at all three levels. The approach will lead to identification of multilevel clusters of risk and factors that facilitate or impede mental health service delivery. The study team will then engage the community partners, and policy and practice advisors to inform development of recommendations to reduce risk and improve mental health for the autistic population.

## Introduction

Suicide is a major and growing public health concern, accounting for an excess of 48,000 deaths annually [1–3]. Suicide behaviors, including suicidal ideation (SI) and suicide attempt (SA), affect over 12 million Americans annually and cost more than $26B annually in medical, work-loss, and quality of life costs [4,5].

One population at increased risk of suicide and suicide behaviors are autistic people (our use of ‘autistic people’ reflects community preferences). Autism is a neurodevelopmental condition defined by differences in social communication and restricted/repetitive behaviors [6], which affects 2.47% of U.S. children and adolescents [7]. Identification of autism has grown annually [8], including for adults overlooked in childhood [9–11]. Epidemiological studies show suicide deaths are 1.5-8 times more likely for autistic than non-autistic people [12–15]. The lifetime pooled prevalence of SI (34.2%; 95% CI: 27.9-40.5%), suicide plans (21.9%; 13.4-30.4%), and SA (24.3%; 18.9-29.6%) among autistic people indicate they are at 7-48 times greater risk than the general population (SI: 4.9%; suicide plans: 1.3%; SA 0.5%) [16–18]. To curb inequities in suicide outcomes for autistic people [12–15,19–22] further research is needed to understand the dynamic influence of individual, community, and policy factors, and to inform policy and practice changes that reflect the autistic community’s needs and priorities [23].

While evidence demonstrates higher risk of suicide behaviors among autistic youth and adults, risk factors are underexplored [15,24,25]. Data, primarily from survey studies, have begun to elucidate a limited set of individual factors associated with elevated suicidality in autistic youth and adults (e.g., sex, gender, age, co-occurring conditions, race). For example, recent studies show autistic females at greater risk for SA and self-harm than autistic males [13,15]. A recent meta-analysis reported that age moderates suicide plans for autistic youth and adults, with higher likelihood in adulthood [18], however only one included study used population-representative data. While most studies of suicide behaviors in autistic people fail to account for racial and ethnic identity, one found increased risk of SI and SA among autistic African American children [26], while three found no significant relationship between race, ethnicity, and risk of suicide behaviors for autistic children and adolescents [26–28]. Co-occurring conditions such as mood and anxiety disorders, substance use disorders, and attention-deficit hyperactivity disorder (ADHD) are consistently found associated with an increased risk of suicide behaviors in autistic youth and adults [13,26,27,29–32]. Even though one-third of autistic people have co-occurring intellectual disability (ID) [33], many previous studies have excluded those with ID [8,18,34,35]. Therefore, the association between co-occurring ID and risk of suicide behaviors is unclear [13,26,29,30].

Despite contemporary models that recognize the influence of social determinants of health on risk of suicide behaviors for the general population [36–38], the influence of community factors has been relatively ignored for autistic youth and adults [18]. Studies with the broader U.S. population have identified that neighborhood and social vulnerabilities, rural contexts, and limited provider availability are associated with increased risk of suicide behaviors [39–45]. While one unique study found that low socioeconomic status (SES) predicted suicide behavior in autistic children [26], there is a substantial need to more closely examine how community factors interact to drive suicide behaviors at a population level. Our understanding of the influence of health policies on risk of suicide behaviors for autistic youth and adults also lags knowledge about the broader population. National studies of Medicaid expansion showed reductions in rates of suicide death among non-elderly adults [46,47], yet it is unclear whether this extends to autistic adults. A recent study showed an increase in Medicaid disenrollment for autistic young adults in states without Medicaid waivers [48], likely limiting their access to healthcare. In the proposed study, we will address these gaps by exploring how individual, community, and policy factors are associated with SI and SA risk among autistic youth and adults by applying data reduction, classification, and machine learning analytic techniques to two integrated, multilevel databases to derive typologies of risk in the autistic population.

Given that access to mental health care predicts suicide behaviors [36,49,50], it is crucial to understand who receives mental health care. Yet again, this is virtually unknown for autistic people. Studies of the U.S. population suggest that individual, community, and policy factors impact receipt of mental health care. African American/Black, Asian, “other” race, and/or Latino individuals have fewer visits (inpatient and outpatient), delay care, and report cost concerns [51–55]. Health policies, such as Medicaid expansion and mental health parity laws increase receipt of mental health care for the broad U.S. population [56,57], although these benefits may not be realized for racial and ethnic minority beneficiaries or those with disabilities [57,58]. Given the increased risk of suicide behaviors for autistic people, there is an immediate need to investigate national healthcare data to drive timely policy changes.

While systematic reviews show that psychotherapeutic interventions including cognitive-behavioral therapy, dialectical behavior therapy, and other psychodynamic approaches effectively reduce suicide behaviors for the general population [59–61], autistic youth and adults have historically been excluded from psychotherapy [62–64]. This has led to reliance on pharmacological therapy (i.e., medications), with unintended consequences of polypharmacy and use of psychotropic medications, often in absence of a corresponding diagnosis [65–67]. A national collaboration to identify mental health needs and priorities for adults with developmental disabilities reported the need for alternatives to pharmacological therapy [68]. However, it remains unknown what socioecological factors are associated with receipt of mental health services, pharmacology, or both for autistic youth and adults. Further, characterizing delivery modality (face-to-face, telemental health, both) for autistic youth and adults is important, given the potential of telemedicine to increase access to mental health care [69]. Collectively, this knowledge could inform policies that enhance receipt of mental health care for autistic people.

There is a clear need for research to identify multilevel factors that could be targeted for multilevel suicide prevention efforts. Evidence shows that multilevel suicide prevention strategies are among the most effective approaches (after structural and means-based interventions, e.g., bridge access restriction, firearms safety) [70]. Yet, there are currently no comprehensive recommendations for suicide prevention policy and practice in the autistic community that address multilevel risk factors [37,71]. The World Health Organization (WHO) has also indicated that effective suicide prevention strategies should involve stakeholders from the outset [70], aligning with broader calls for research involvement from the disability community (i.e., “nothing about us without us” [71]).

To reduce the risk of suicide behaviors and other negative mental health outcomes for the autistic population, this study examines multilevel, socioecological factors associated with suicide behaviors and mental health service receipt [72]. Using a sequential, mixed methods design [33], the approach incorporates collaboration with autistic stakeholders and policy and practice consultants across three aims to support relevance and translation of findings.

Aim 1: Identify clusters of socioecological factors (at individual, community, and policy levels) associated with risk of SI and SA among two national samples of autistic youth and adults.
Aim 2: Evaluate socioecological factors associated with receipt of mental health care (mental health service, pharmacology, both, neither), dose (visits/year), and delivery modality (face-to-face, telemental health, both) among two national samples of autistic youth and adults with documented suicide behaviors and/or co-occurring mental health conditions.
Exploratory Aim 3: Develop evidence-based, community-driven recommendations to reduce risk of suicide behaviors and facilitate receipt of mental health services for autistic youth and adults (not discussed here).

## Materials and methods

### Consultation

In alignment with the WHO’s recommendations to involve stakeholders, we will engage with the established Academic Autism Spectrum Partnership in Research and Education (AASPIRE) Suicide Prevention Project (SPP) team of autistic community research partners. The AASPIRE SPP team was launched in 2022 as part of the larger AASPIRE organization to conduct community-engaged research on autism and suicide prevention. AASPIRE is a well-established, long-standing community-based participatory research collaboration that has conducted projects on healthcare, mental health, employment, and measurement since 1996 [73,74]. The AASPIRE SPP team’s expertise offers insight based on community member experiences to maximize community relevance and to support translation. AASPIRE SPP team members have personal and professional experiences relevant to the project aims. They represent autistic community diversity in terms of age, gender, race/ethnicity, LGBTQIA + identity, region of the U.S., experience with private and public health insurance, experience accessing mental health services, co-occurring conditions, communication (i.e., speaking and non-speaking), and needs. The AASPIRE SPP team will meet approximately four times per year of the project to consult on Aims 1 and 2 variable selection, preliminary findings, interpretation, and to address problems as they arise. For Exploratory Aim 3, they will assist with identifying key information to share in focus groups, developing focus group materials, and interpreting focus group results.

We will also rely on expertise from a council of policy and practice advisors with expertise relevant to the project aims. These Policy and Practice Advisory Council members have expertise in psychiatry, suicide prevention, mental health and developmental disability/autism policies, Medicaid, and healthcare reimbursement. They will provide input annually across all aims to ensure findings can be optimally leveraged to inform policy and practice. Both AASPIRE SPP and the policy and practice advisors will assist with the development and finalization of recommendations as the ultimate outcome of the study (in Exploratory Aim 3, not discussed here).

### Aims 1 and 2

#### Ethics statement.

The University of Florida IRB deemed this study as exempt and approved through protocol IRB202401169, including waiver of informed consent.

#### Data.

Aims 1 and 2 will integrate sources containing individual, community, and policy-level information. The sources that comprise each level are outlined below.

*Individual:* Individual-level data will be extracted from two national samples of health insurance claims between October 1, 2015 and December 31, 2022: 1) IBM® MarketScan® Research Database (MarketScan®) and 2) Centers for Medicare & Medicaid Services (CMS) MAX & MSIS (Medicaid). 1) MarketScan is a research data set that integrates deidentified patient-level health data, hospital discharges, and electronic health records. Data are contributed by large employers, managed care organizations, hospitals, and electronic health record providers across the continuum of care [75]. The database contains detailed prescription drug information and indicates the type, timing, and condition/diagnosis related to each healthcare interaction, as well as beneficiaries’ three-digit zip code. 2) Each state’s Medicaid Management Information System collects enrollment and claims data for persons enrolled in Medicaid and the Children’s Health Insurance Program. This information is standardized into a consistent, national format by the Medicaid Statistical Information System (MSIS). To support research and policy analysis initiatives for Medicaid and other low-income populations, MSIS generates a set of person-level data files on Medicaid eligibility, service utilization, and payments called the Medicaid Analytic eXtract (MAX). The claims in MAX identify the services rendered, cost of those services, and nine-digit zip code of the beneficiary for each calendar year indicating the type, timing, and condition/diagnosis related to all health system interactions.

*Community:* The Social Vulnerability Index (SVI) [76], developed by Cutter et al. [77], indicates the relative vulnerability of every U.S. Census tract by ranking 16 social factors and grouping them into four related themes. SVI is updated every two years, and the four themes provide socially and spatially relevant information that allow for multi-state mapping and analysis. Tracts are also ranked for the entire U.S. based on percentiles, with higher values indicating greater vulnerability. Each tract receives a rank for the 1) individual component variables, 2) four themes, and 3) overall position. This data measures aspects of social vulnerability at the Census tract level. Additionally, the Health Resources and Services Administration Area Health Resource Files (AHRF) contains over 6,000 variables related to healthcare access at the county and city levels (e.g., health providers, health facility numbers and types, hospital utilization, population characteristics, economic data). These data will capture mental health providers and infrastructure in the communities in which autistic youth and adults live.

*Policy:* Since January 1, 2014, states have had the option to extend Medicaid coverage to most non-elderly adults with income up to 138% of the federal poverty line (FPL). However, states varied significantly in their timing of expansion and implementation as well as their use of Section 1115 waiver stipulations, extensions, and amendments (i.e., regulatory allowances for change in state program eligibility, benefits, provider payments, and other rules). To capture the chronology of state-level changes made during the transition to full Medicaid expansion, the Kaiser Family Foundation developed an archival data base of all state-level Medicaid expansion and implementation information including dates, waiver stipulations, and changes in coverage. These data will allow us to determine the association between the type and timing of Medicaid expansion and observed outcomes [47]. Since mental health insurance regulations vary across states and time, the State Mental Health Insurance Laws (SMHIL) Dataset [78] tracks state legal characteristics across six domains—parity, coverage, definition, all conditions defined, enforcement, and compliance. Collected since 1997, SMHIL employs rigorous policy surveillance methodology to track changes in state mental health insurance laws.

*Integration:* The integration of Medicaid Expansion, SMHIL, SVI, and AHRF with the MarketScan and Medicaid claims files will use individual zip codes in the claims, Federal Information Processing Standards (FIPS) codes in the AHRF file, and census tract identifiers in the SVI database. To match the state-level information in the Medicaid Policies database and the SMHIL Dataset, we will use annual lists of state-level zip code ranges which are published by the Internal Revenue Service and U.S. Postal Service (USPS). This publicly available information will allow the state location of each zip code to be easily identified and matched. State-level and county FIPS codes in the AHRF file will be converted to zip code ranges using the CDC County Cross Reference File (FIPS/ZIP4). Finally, SVI data comprises Census tract identifiers. To link individual Census tracts with zip codes, we will utilize the US Department of Housing and Urban Development (HUD) Office of Policy Development and Research HUD-USPS ZIP Code Crosswalk data which is published quarterly. This information originates directly from the USPS making it highly responsive to changes in zip code configuration.

#### Inclusion/Exclusion criteria.

This study focuses on autistic youth and adults between the ages of 12 (consistent with the age minimums in important national surveys of youth, e.g., the National Longitudinal Survey of Youth and the National Longitudinal Study of Adolescent to Adults Health) and 64 (since we do not utilize Medicare data) as of October 1, 2015 (following the conversion to ICD-10). Consistent with existing literature, an individual will be classified as autistic [79–81] if they have ≥ 1 inpatient claim or ≥ 2 other service claims with an autism diagnosis code in any year. Individuals without an identifiable birth date and at least 12-months of continuous enrollment will be excluded.

#### Key outcomes.

*Aim 1, Suicide behaviors:* SI will be identified by presence of ICD-10 code R45.851 for an inpatient stay or outpatient visit, while SA will be captured by ICD-10-CM code T14.91. Given distinct differences between SI and SA, these outcomes will be analyzed separately in both the MarketScan and Medicaid databases. Annual aggregates for SI and SA will be created for individuals with more than one related claim during a calendar year. The medical claims data will be used to create a data set consisting of person-year records. Using the 2015-to-2022 timeframe, individuals may have up to eight records if they were continuously enrolled for all eight years. A total count of the number of SI or SA events during a given year will be created to form a longitudinal panel each (one for SI/one for SA) with up to eight observations per person. For example, an autistic individual who was available during all years and did not have a SI will have eight annual observations with an event code of 0 for all records. An individual who had his/her coverage revoked after 3 years and did not have any SI would have three annual observations with an event code of 0 for all three. An individual who had one SI in year 4 would have a total of four records with event codes of 0 for the first 3 years and a code of 1 on the fourth year. Prior SI will be included as a lagged covariate in the model. This will be repeated for SA.

*Aim 2, Receipt of mental health services:* Current Procedural Terminology (CPT) and Healthcare Common Procedure Coding System (HCPCS) codes will indicate the receipt of mental health services. Dose of mental health services will be quantified as the number of visits per calendar year received among those with any observed visits. Since mental health services are often coupled with pharmacological therapies, making their independent effects inextricable, we will create a categorical variable with four mutually exclusive categories describing treatment received: pharmacology only, mental health services only, both pharmacology and mental health services, and no treatment.

*Receipt of pharmacological therapy:* Pharmacological therapies will be grouped using claims data (prescription days of supply) into eight common classes: antidepressant, antipsychotic, mood stabilizers, stimulants, anxiolytics, central nervous system depressants, substance abuse medications, and cognitive enhancers. A drug will be considered present during a given month if a prescription was filled in that month or a carryover from a prior month was filled (i.e., day’s supply extended into the next month). In cases where a prescription was filled in the month of a SI or SA code, the drug was considered present if the prescription was filled prior to the event.

#### Key covariates.

*Individual:* The primary way to classify phenotypic differences within the autistic population through secondary data analysis is examination of co-occurring diagnoses [82], including three categories of conditions—mental health conditions, substance use disorder, and developmental conditions. Mental health conditions will be identified in claims using ICD-10 codes. Rather than dichotomizing the high number of specific mental health diagnoses, binary indicators will be created indicating the mental health condition category. The categorical framework is based on the phenome-wide association study (PheWAS) [83] and includes psychosis disorders, mood disorders, anxiety disorders, personality disorders, psychogenic and somatoform disorders, adjustment disorders, eating disorders, and other psychological disorders. Substance use disorder, indicated as important in prior studies [84–86] but not distinguished in the PheWAS, will be identified in claims using ICD-10 codes. Substance use disorder will be dichotomized. Developmental conditions (ID and ADHD) will be identified in claims using ICD-10 codes. Each condition with be dichotomized. Additional individual-level information will include age, sex, and race/ethnicity.

*Community:* SVI groups 16 social factors obtained from Census variables into four themes—SES, household composition and disability, minority status and language, and housing type and transportation. Each county is assigned a percentile ranking for each theme as well as an overall composite score. To evaluate both the overall vulnerability as well as individual themes, we will first include the overall SVI composite score in the analytic model, then substitute the individual theme scores to test the overall sensitivity of each area. AHRF data extracted will include area rurality (captured by the Rural–Urban Commuting Area Codes), accessibility of healthcare centers/facilities, and availability of primary care and specialty providers.

*Policy factors:* Six aspects of state-level Medicaid expansion will be included in the analysis: 1) Number of months since adoption of the Affordable Care Act Medicaid expansion, 2) Variation in adult/child income threshold (if different from 138% FPL), 3) Capitated enrollment, 4) Benefit limitations, 5) Work requirements, and 6) Central mechanism (premium assistance or Medicaid program). The SMHIL captures six characteristics of state mental health insurance laws using a categorical coding scheme and assigns each state an annual score based on these characteristics. Annual state scores will be included in the model. To test the sensitivity of estimates, annual scores will be replaced by its six components to determine variation in policy impact on outcomes. The declaration of a public health emergency in 2020 and a nation-wide effort to stop the spread of COVID-19 shifted patterns of insurance coverage, healthcare delivery, and reimbursement. Various state and federal legislation mandated that most public and private insurers extended coverage to telehealth and other remote care services. While these regulatory changes will be captured in the SMHIL and Medicaid expansion databases, we will also consider the disproportionate impact of pandemic-related changes on autistic individuals through the inclusion of sensitivity testing and additional, pandemic-focused analysis to evaluate these unique circumstances.

#### Sample size considerations.

While the prevalence of SI and SA among autistic individuals has never been assessed using large, nationally representative data sets like those proposed here, U.S.-based studies have estimated that SI is between 11% and 22% while SA is between 1% and 7%. Based on these existing prevalence estimates, a power analysis was conducted using R software package *mpower* [87] to determine a sample range that would be required to observe statistically significant differences if they exist. Using a power of 0.8, an alpha of 0.05, and a Z_α/2_ of 1.96, a sample of SI between 2,653 and 6,053 and a sample of SA between 161 and 252 would be required based on these proportions. Between 2015 and 2018, suicide behaviors meeting the claims coding requirements were observed an average of 4,599 (SI) and 252 (SA) times in MarketScan, and 2,346 (SI) and 481 (SA) times in Medicaid claims, suggesting a sufficient sample size in both data sources.

#### Aim 1 statistical analysis approach.

Using ΣSIit, ΣSAit as dependent variables in analyses of SI and SA, respectively, analyses of SI and SA will first use multiple correspondence analysis (MCA) to reduce dimensionality then employ hierarchical clustering analysis (HCA) with internal cross validation for estimation. MCA is a data analysis technique for categorical data, used to detect and represent underlying structures on a Burt table—a symmetrical, generalized contingency table of all two-way cross-tabulations between the categorical variables analogous to the covariance matrix of continuous variables [88]. HCA is a machine learning procedure that classifies individuals with similar profiles and contexts into distinct groups based on a set of measured variables without imposing existing assumptions or biases. Internal cross validation verifies the predictive ability of the model by randomly dividing the data into subsets and iteratively utilizing subsets for training and validating to ensure the optimal number of clusters.

MCA is a principal component analysis method that transforms categorical data into coordinates in multidimensional space and outputs several dimensional solutions. MCA yields a “space” that summarizes the associations between individual, community, and policy factors. HCA of the MCA coordinates will be applied using Ward minimum-variance linkage to assign individuals into distinct groups according to their annual SI and SA patterns. This method starts with everyone in their own cluster, combining the most “similar” individuals based on closeness in Euclidean distance and continuing until the last 2 clusters are merged into 1 cluster containing all participants. To determine the optimal number of clusters [89] we will apply seven clustering methods (K-means, partition around medoids [PAM], fuzzy K-means, probabilistic distance clustering [PDClust], mixture of multivariate normal distributions [MVN], mixture of skewed-t distributions [MST], and mixture generalized hyperbolic distributions [MGHD]) to the transformed data set from MCA. We will use cluster comparison metrics such as the Calinski-Harabasz criterion [90], Bayesian Information criterion (BIC) [91], and Adjusted Rand Index (ARI) [92] to analyze and compare clustering results. Then the optimal number of clusters will be selected by calculating the sum of the within-cluster inertia at each partition, and partitioning clusters [93] where there is the highest relative loss of inertia [94]. Characteristics (individual, community, and policy variables) will be compared between clusters and internal cross validation with iterative subsets will be used to further determine the convergence and validity of the clustering results. HCA will yield a group of cluster membership variables reflecting the partitioning of individuals into homogeneous groups based on selected characteristics. The contribution of each characteristic to the group formation is indicated to reveal a typology of SI and SA clusters.

#### Aim 2 statistical analysis approach.

**Fig 1:**
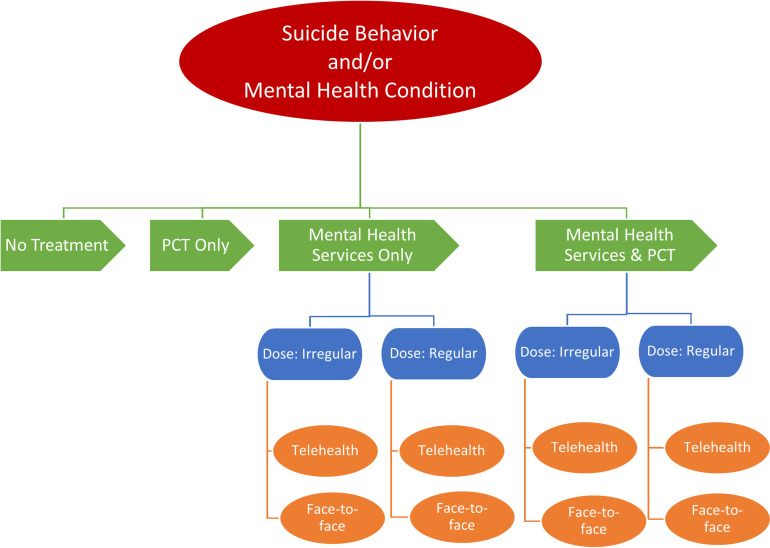
Aim 2 Outcomes. The sequential order of outcomes for the Aim 2 study population of autistic people with a suicide behavior or mental health condition.

A sample of individuals with autism and either (1) ΣSIit, > 0 or ΣSAit > 0, or (2) mental health conditions > 0 will be used to assess receipt of mental health services (mental health services, pharmacology, both, and neither), dose (visits/year), and delivery modality (face-to-face, telemental health, both). As the picture in Fig 1 shows, the sequential order of outcomes begins with Tier I—receipt of mental health care—and will utilize four mutually exclusive categorical dependent variables: pharmacological therapy only, mental health services only, both pharmacology and mental health services, and no treatment. Tier II—dose of mental health services receipt—will be quantified as the number of visits per calendar year received among those with any observed visits. Given insurance coverage stipulations, “regular” therapy will be considered eight or more sessions within a calendar year. Tier III—type of mental health service delivery—will indicate the mode of delivery as face-to-face, telemental health, or both.

The data analysis will involve three primary stages. First, to reduce data dimensionality we will conduct MFPCA [95,96]— a statistical methodology designed to extract core intra- and inter-subject geometric components of multilevel data. To ensure that the optimal number of components are retained, cross validation using randomly partitioned data subsets will be conducted to optimize the mean squared error (MSE)/area under the curve (AUC) as appropriate for discrete/binary outcomes. Second, multilevel sequential logit models [97] (SLM) will evaluate factors associated with mental health care receipt, dose, and delivery modality. Finally, we will evaluate the sensitivity of the analysis to age, race, and sex to determine the potential influence on the relationship between the factors and receipt, dose, and modality of mental health care.

A SLM [97] is an appropriate method to analyze these sequential data since SLM [97] assumes that decisions are not made simultaneously, but rather in a specific order. Tier I will be estimated with a multinomial logistic regression, while Tiers II and III will be estimated with binary logistic regressions. Since factors at multiple levels (individual, community, policy) are associated with receipt, dose, and delivery of mental health services, we will use multilevel SLM to account the nested structure of the data. To understand the influence of individual, community, and policy factors, the analysis will be structured such that we begin with a basic, parsimonious model specification and add factors successively as we increase the model complexity.

The intra-class correlation (ICC) between random variables will be calculated for variables in each of the three multilevel models [98]. We will verify conditions of stationarity and homogeneity within the data and evaluate model fit to ensure appropriate specification and robust findings. We will also provide odds ratios (ORs) and interval odds ratios [99] (IORs) which quantify the effect of cluster‐level variables when using multilevel logistic regression models.

### Exploratory Aim 3

To develop evidence-based, community-driven recommendations to reduce risk of suicide behaviors and facilitate receipt of mental health services for autistic youth and adults, this study incorporates a qualitative focus group approach that sequentially follows Aims 1 and 2. The study team will engage the experts from the AASPIRE SPP and the Policy and Practice Advisory Council to inform collection of focus group data from a larger group of community stakeholders including autistic adults (n =  25), family caregivers (n =  25), and mental health clinicians (n =  15).

#### Aim 3 procedures.

Engaging community members in the interpretation of research findings to inform action is an established participatory research methodology [100]. To identify critical findings and generate preliminary recommendations, the research team will first distill and integrate key findings from the Medicaid and MarketScan analyses and prioritize findings that reveal re-occurring patterns of individual, community, and policy factors associated with low/high risk of suicide behaviors and receipt of mental health services [101]. The team will also look for differences or similarities in clusters of risk and receipt of mental health care across insurance type and then: 1) Review our distilled patterns of findings with the AASPIRE SPP and discuss priorities for which results may have the greatest community importance; 2) Present the initial patterns of findings as well as AASPIRE SPP’s priorities at the Policy and Practice Advisory Council annual meeting to gather feedback on which findings have the greatest potential for policy and practice recommendations and to generate preliminary recommendations; and 3) Return to the AASPIRE SPP to make integrated decisions about what findings will be shared in the focus groups and to synthesize preliminary recommendations to be used in the focus groups, to be reported elsewhere.

### Study timeline

This four-year project commenced with the record screening phase in August 2024. Data extraction and analysis for Aims 1 and 2 will occur through the third year of the four-year project. Results are expected during the second and third years for Aim 1, followed by results for Aim 2 during the third and fourth years. Participant recruitment and focus groups for Exploratory Aim 3 (not discussed here) will initiate during the third year, with data analysis completing and results expected the early portion of the fourth year.

## Discussion

This study will identify and characterize individual, community, and policy risk factors associated with suicide behaviors and receipt of mental health services among individuals with autism. By identifying multiple levels of socioecological factors with mutual, comingled associations with risk of suicidal ideation and suicide attempts, this study will produce translatable findings that, through integration with lived-experience expertise, will become actionable recommendations for reducing risk of suicide behaviors and improving mental health care for autistic people. This study will demonstrate significant progress towards reducing risk of suicide behaviors in the autistic community and supporting improved mental health policy and practice.

### Limitations

This study is not designed to directly attribute the observed suicide behaviors to specific individual, community, or policy factors, but rather show the strength of the associations between SI, SA, and these attributes. Despite efforts to account for heterogeneity, we acknowledge that additional unobserved differences likely exist. Therefore, all estimates will be reported within the context in which they are derived. This study utilizes healthcare claims data are subject to some degree of missingness, both systematic and otherwise. Records missing pivotal outcomes (e.g., date of birth, diagnoses, enrollment type) will be excluded. To ensure that missing data only introduces random variation, we will attempt to impute missing data using a regression switching approach and conduct a sensitivity analysis with imputed data. However, we will carefully examine missing data to ensure no systematic patterns are observed and confer with AASPIRE SPP and the Policy and Practice Advisory Council for additional insights and recommendations. To ensure that model selection does not bias results, we will adopt an objective approach for study sample selection. Decisions to limit the range of data used in analyses will be reported transparently. We will incorporate sensitivity analyses, robustness tests, and adherence to methodological and statistical rigor [102–110]. Furthermore, we will engage AASPIRE SPP to assist with interpretation of study findings to ensure relevance. Finally, state-to-state variation in Medicaid programs and expansion rates may result in heterogeneous covered populations, thereby obscuring national-level generalizations. If cross-state differences in recipient sex, age, race, and income profiles present sample biases that confound the proposed analysis, we will segment states into four groups—early, middle, late, and no expansion—following the framework of Lee, et al. [111]. The early, middle, late, and no expansion clusters will be included as a stratum in the analysis of Medicaid claims as well as estimated as separate entities.

## Conclusion

This study will identify and characterize individual, community, and policy risk factors associated with suicide behaviors and receipt of mental health services among autistic individuals. By identifying multiple levels of socioecological factors with mutual, comingled associations with suicide behaviors risk, we will produce translatable findings that, through community informed dialogue, will translate to actionable recommendations for reducing risk of suicide behaviors and improving mental health care for autistic people. Our team’s broad network of mental health, autistic, and clinical associates and organizations provides a diverse audience for this project’s findings and resulting recommendations. This study will demonstrate significant progress toward informing our long-term goal of reducing risk of suicide behaviors in the autistic community.

## References

[1] National Action Alliance for Suicide Prevention: Research Prioritization Task Force. A prioritized research agenda for suicide prevention: an action plan to save lives. Rockville (MD): National Institute of Mental Health and the Research Prioritization Task Force; 2014 [cited 2014]. Available from: https://theactionalliance.org/sites/default/files/agenda.pdf.

[2] Hedegaard H, Curtin SC, Warner M. Increase in suicide mortality in the United States, 1999-2018. Hyattsville (MD): National Center for Health Statistics; 2020. 32487287

[3] Centers for Disease Control and Prevention. Suicide data and statistics 2023. Available from: https://www.cdc.gov/suicide/facts/data.html?CDC_AAref_Val=https://www.cdc.gov/suicide/suicide-data-statistics.html.

[4] Centers for Disease Control and Prevention, National Center for Injury Prevention and Control. Web-based Injury Statistics Query and Reporting System (WISQARS), cost of injury reports. In: Centers for Disease Control and Prevention NCfIPaC, editor. Atlanta (GA): Centers for Disease Control and Prevention, National Center for Injury Prevention and Control.

[5] Office of the Assistant Secretary for Planning and Evaluation. Guidelines for regulatory impact analysis. U.S. Department of Health and Human Services; 2016.

[6] American Psychological Association. Diagnostic and statistical manual of mental disorders. 5th ed. Washington (DC): American Psychological Association; 2013.

[7] Xu G, Strathearn L, Liu B, Bao W. Prevalence of autism spectrum disorder among US children and adolescents, 2014-2016. JAMA. 2018;319(1):81–2. doi: 10.1001/jama.2017.17912 29297068 PMC5833544

[8] Maenner M, Warren Z, Williams A, Amoakohene E, Bakian A, Bilder D. Prevalence and characteristics of autism spectrum disorder among children aged 8 years - Autism and developmental disabilities monitoring network, 11 sites, United States, 2020. MMWR Surveill Summ. 2023;72(2):1–14. 10.15585/mmwr.ss7202a1 PMC10042614 36952288

[9] Keller R, Chieregato S, Bari S, Castaldo R, Rutto F, Chiocchetti A, et al. Autism in adulthood: clinical and demographic characteristics of a cohort of five hundred persons with autism analyzed by a novel multistep network model. Brain Sci. 2020;10(7):416. doi: 10.3390/brainsci10070416 32630229 PMC7407178

[10] Dietz PM, Rose CE, McArthur D, Maenner M. National and state estimates of adults with autism spectrum disorder. J Autism Dev Disord. 2020;50(12):4258–66. doi: 10.1007/s10803-020-04494-4 32390121 PMC9128411

[11] Lai M-C, Baron-Cohen S. Identifying the lost generation of adults with autism spectrum conditions. Lancet Psychiatry. 2015;2(11):1013–27. doi: 10.1016/S2215-0366(15)00277-1 26544750

[12] Kirby AV, Bakian AV, Zhang Y, Bilder DA, Keeshin BR, Coon H. A 20-year study of suicide death in a statewide autism population. Autism Res. 2019;12(4):658–66. doi: 10.1002/aur.2076 30663277 PMC6457664

[13] Hirvikoski T, Boman M, Chen Q, D’Onofrio BM, Mittendorfer-Rutz E, Lichtenstein P, et al. Individual risk and familial liability for suicide attempt and suicide in autism: a population-based study. Psychol Med. 2020;50(9):1463–74. doi: 10.1017/S0033291719001405 31238998

[14] Hirvikoski T, Mittendorfer-Rutz E, Boman M, Larsson H, Lichtenstein P, Bölte S. Premature mortality in autism spectrum disorder. Br J Psychiatry. 2016;208(3):232–8. doi: 10.1192/bjp.bp.114.160192 26541693

[15] Kõlves K, Fitzgerald C, Nordentoft M, Wood SJ, Erlangsen A. Assessment of suicidal behaviors among individuals with autism spectrum disorder in Denmark. JAMA Netw Open. 2021;4(1):e2033565. doi: 10.1001/jamanetworkopen.2020.33565 33433599 PMC12578491

[16] Substance Abuse and Mental Health Services Administration. Key substance use and mental health indidcators in the United States: results from the 2020 National Survey on Drug Use and Health (HHS Publication No. PEP21-07-01-003, NSDUH Series H-56). Rockville (MD): Center for Behavioral Health Statistics and Quality; 2021.

[17] Substance Abuse and Mental Health Services Administration. Key substance use and mental health indicators in the United States: Results from the 2019 National Survey on Drug Use and Health (HHS Publication No. PEP20-07-001. Rockville (MD); 2020.

[18] Newell V, Phillips L, Jones C, Townsend E, Richards C, Cassidy S. A systematic review and meta-analysis of suicidality in autistic and possibly autistic people without co-occurring intellectual disability. Mol Autism. 2023;14(1):12. doi: 10.1186/s13229-023-00544-7 36922899 PMC10018918

[19] Cassidy S, Bradley P, Robinson J, Allison C, McHugh M, Baron-Cohen S. Suicidal ideation and suicide plans or attempts in adults with Asperger’s syndrome attending a specialist diagnostic clinic: a clinical cohort study. Lancet Psychiatry. 2014;1(2):142–7. doi: 10.1016/S2215-0366(14)70248-2 26360578

[20] Chen M-H, Pan T-L, Lan W-H, Hsu J-W, Huang K-L, Su T-P, et al. Risk of suicide attempts among adolescents and young adults with autism spectrum disorder: a nationwide longitudinal follow-up study. J Clin Psychiatry. 2017;78(9):e1174–9. doi: 10.4088/JCP.16m11100 28872268

[21] Croen LA, Zerbo O, Qian Y, Massolo ML, Rich S, Sidney S, et al. The health status of adults on the autism spectrum. Autism. 2015;19(7):814–23. doi: 10.1177/1362361315577517 25911091

[22] Segers M, Rawana J. What do we know about suicidality in autism spectrum disorders? A systematic review. Autism Res. 2014;7(4):507–21. doi: 10.1002/aur.1375 24798640

[23] Kramer JM, Schwartz AE, Watkins D, Peace M, Luterman S, Barnhart B, et al. Improving research and practice: priorities for young adults with intellectual/developmental disabilities and mental health needs. J Ment Health Res Intellect Disabil. 2019;12(3–4):97–125. doi: 10.1080/19315864.2019.1636910

[24] Cassidy S, Rodgers J. Understanding and prevention of suicide in autism. Lancet Psychiatry. 2017;4(6):e11. doi: 10.1016/S2215-0366(17)30162-1 28551299

[25] Camm-Crosbie L, Bradley L, Shaw R, Baron-Cohen S, Cassidy S. “People like me don’t get support”: autistic adults’ experiences of support and treatment for mental health difficulties, self-injury and suicidality. Autism. 2019;23(6):1431–41. doi: 10.1177/1362361318816053 30497279 PMC6625034

[26] Mayes SD, Gorman AA, Hillwig-Garcia J, Syed E. Suicide ideation and attempts in children with autism. Res Autism Spectr Disord. 2013;7(1):109–19. doi: 10.1016/j.rasd.2012.07.009

[27] Storch EA, Sulkowski ML, Nadeau J, Lewin AB, Arnold EB, Mutch PJ, et al. The phenomenology and clinical correlates of suicidal thoughts and behaviors in youth with autism spectrum disorders. J Autism Dev Disord. 2013;43(10):2450–9. doi: 10.1007/s10803-013-1795-x 23446993 PMC3808993

[28] Horowitz LM, Thurm A, Farmer C, Mazefsky C, Lanzillo E, Bridge JA, et al. Talking about death or suicide: prevalence and clinical correlates in youth with autism spectrum disorder in the psychiatric inpatient setting. J Autism Dev Disord. 2018;48(11):3702–10. doi: 10.1007/s10803-017-3180-7 28624965 PMC7410502

[29] McDonnell CG, DeLucia EA, Hayden EP, Anagnostou E, Nicolson R, Kelley E, et al. An exploratory analysis of predictors of youth suicide-related behaviors in autism spectrum disorder: implications for prevention science. J Autism Dev Disord. 2020;50(10):3531–44. doi: 10.1007/s10803-019-04320-6 31820342

[30] Holden R, Mueller J, McGowan J, Sanyal J, Kikoler M, Simonoff E, et al. Investigating bullying as a predictor of suicidality in a clinical sample of adolescents with autism spectrum disorder. Autism Res. 2020;13(6):988–97. doi: 10.1002/aur.2292 32198982 PMC8647922

[31] Dell’Osso L, Carpita B, Muti D, Morelli V, Salarpi G, Salerni A, et al. Mood symptoms and suicidality across the autism spectrum. Compr Psychiatry. 2019;91 34–8. doi: 10.1016/j.comppsych.2019.03.004 31003723

[32] Hooijer AAT, Sizoo BB. Temperament and character as risk factor for suicide ideation and attempts in adults with autism spectrum disorders. Autism Res. 2020;13(1):104–11. doi: 10.1002/aur.2221 31622053

[33] Castro FG, Kellison JG, Boyd SJ, Kopak A. A methodology for conducting integrative mixed methods research and data analyses. J Mix Methods Res. 2010;4(4):342–60. doi: 10.1177/1558689810382916 22167325 PMC3235529

[34] Maenner M, Shaw K, Bakian A, Bilder D, Durkin M, Esler A, et al. Prevalence and characteristics of autism spectrum disorder among children aged 8 years - Autism and developmental disabilities monitoring network, 11 sites, United States, 2018. MMWR Surveill Summ. 2021;70(11):1–16. doi: 10.15585/mmwr.mm7011a1 PMC8639024 34855725

[35] Maenner M, Shaw K, Baio J, , Washington A, Patrick M, et al. Prevalence of autism spectrum disorder among children aged 8 years - Autism and developmental disabilities monitoring network, 11 sites, United States, 2016. MMWR Surveill Summ. 2020;69(4):1–12. doi: 10.15585/mmwr.ss6904a1 PMC7119644 32214087

[36] Turecki G, Brent DA. Suicide and suicidal behaviour. Lancet. 2016;387(10024):1227–39. doi: 10.1016/S0140-6736(15)00234-2 26385066 PMC5319859

[37] Standley CJ. Expanding our paradigms: Intersectional and socioecological approaches to suicide prevention. Death Stud. 2022;46(1):224–32. doi: 10.1080/07481187.2020.1725934 32048555

[38] Standley CJ, Foster-Fishman P. Intersectionality, social support, and youth suicidality: a socioecological approach to prevention. Suicide Life Threat Behav. 2021;51(2):203–11. doi: 10.1111/sltb.12695 33876493

[39] Mohatt NV, Kreisel CJ, Hoffberg AS, Mph LW, Beehler SJ. A systematic review of factors impacting suicide risk among rural adults in the United States. J Rural Health. 2021;37(3):565–75. doi: 10.1111/jrh.12532 33210399

[40] Liu S, Morin S, Bourand N, DeClue I, Delgado G, Fan J. Social vulnerability and risk of suicide in US adults, 2016-2020. JAMA Network Open. 2023;6(4):e239995. 37099296 10.1001/jamanetworkopen.2023.9995 PMC10134005

[41] Sugg MM, Runkle JD, Andersen LM, Desjardins MR. Exploring place-based differences in suicide and suicide-related outcomes among North Carolina adolescents and young adults. J Adolesc Health. 2023;72(1):27–35. doi: 10.1016/j.jadohealth.2022.06.013 35985915

[42] Wang J, Brown MM, Ivey-Stephenson AZ, Xu L, Stone DM. Rural-urban comparisons in the rates of self-harm, U.S., 2018. Am J Prev Med. 2022;63(1):117–20. doi: 10.1016/j.amepre.2021.12.018 35249778 PMC9718505

[43] Steelesmith DL, Fontanella CA, Campo JV, Bridge JA, Warren KL, Root ED. Contextual factors associated with county-level suicide rates in the United States, 1999 to 2016. JAMA Netw Open. 2019;2(9):e1910936. doi: 10.1001/jamanetworkopen.2019.10936 31490540 PMC6735416

[44] Xi W, Banerjee S, Olfson M, Alexopoulos GS, Xiao Y, Pathak J. Effects of social deprivation on risk factors for suicidal ideation and suicide attempts in commercially insured US youth and adults. Sci Rep. 2023;13(1):4151. doi: 10.1038/s41598-023-31387-0 36914764 PMC10011396

[45] Cook BL, Zuvekas SH, Chen J, Progovac A, Lincoln AK. Assessing the individual, neighborhood, and policy predictors of disparities in mental health care. Med Care Res Rev. 2017;74(4):404–30. doi: 10.1177/1077558716646898 27147641 PMC5865608

[46] Austin AE, Naumann RB, Short NA. Association between medicaid expansion and suicide mortality among nonelderly US adults. Am J Epidemiol. 2021;190(9):1760–9. doi: 10.1093/aje/kwab130 34467410 PMC12931419

[47] Patel H, Barnes J, Osazuwa-Peters N, Bierut LJ. Association of state Medicaid expansion status with rates of suicide among US adults. JAMA Netw Open. 2022;5(6):e2217228. doi: 10.1001/jamanetworkopen.2022.17228 35704315 PMC9201676

[48] Carey ME, Tao S, Koffer Miller KH, Marcus SC, Mandell DS, Epstein AJ, et al. Association between Medicaid waivers and Medicaid disenrollment among autistic adolescents during the transition to adulthood. JAMA Netw Open. 2023;6(3):e232768. doi: 10.1001/jamanetworkopen.2023.2768 36912840 PMC10011936

[49] Steele IH, Thrower N, Noroian P, Saleh FM. Understanding suicide across the lifespan: a United States perspective of suicide risk factors, assessment & management. J Forensic Sci. 2018;63(1):162–71. doi: 10.1111/1556-4029.13519 28639299

[50] Yang K, Rodgers CRR, Lee E, Le Cook B. Disparities in mental health care utilization and perceived need among Asian Americans: 2012-2016. Psychiatr Serv. 2020;71(1):21–7. 31575351 10.1176/appi.ps.201900126

[51] Novak P, Anderson AC, Chen J. Changes in health insurance coverage and barriers to health care access among individuals with serious psychological distress following the affordable care act. Adm Policy Ment Health. 2018;45(6):924–32. doi: 10.1007/s10488-018-0875-9 29754279 PMC6477535

[52] McMorrow S, Kenney GM, Long SK, Goin DE. Medicaid expansions from 1997 to 2009 increased coverage and improved access and mental health outcomes for low-income parents. Health Serv Res. 2016;51(4):1347–67. 26762198 10.1111/1475-6773.12432 PMC4946033

[53] Olfson M, Zuvekas SH, McClellan C, Wall MM, Hankerson SH, Blanco C. Racial-ethnic disparities in outpatient mental health care in the United States. Psychiatr Serv. 2023;74(7):674–83. doi: 10.1176/appi.ps.20220365 36597696

[54] Biener A, Zuvekas S. Do racial and ethnic disparities in mental health treatment vary with underlying mental health? Med Care Res Rev. 2021;78(4):392–403. 32028834 10.1177/1077558720903589

[55] Heboyan V, Douglas M, McGregor B, Benevides T. Impact of mental health insurance legislation on mental health treatment in a longitudinal sample of adolescents. Medical Care. 2021;59(10):939–46. 34369459 10.1097/MLR.0000000000001619 PMC8425633

[56] Lee H, Porell FW. The effect of the affordable care act medicaid expansion on disparities in access to care and health status. Med Care Res Rev. 2020;77(5):461–73. doi: 10.1177/1077558718808709 30362848

[57] Creedon TB, Zuvekas SH, Hill SC, Ali MM, McClellan C, Dey JG. Effects of Medicaid expansion on insurance coverage and health services use among adults with disabilities newly eligible for Medicaid. Health Serv Res. 2022;57 Suppl 2(Suppl 2):183–94. doi: 10.1111/1475-6773.14034 35811358 PMC9660429

[58] Sobanski T, Josfeld S, Peikert G, Wagner G. Psychotherapeutic interventions for the prevention of suicide re-attempts: a systematic review. Psychol Med. 2021;51(15):2525–40. doi: 10.1017/S0033291721003081 34608856

[59] Mann JJ, Michel CA, Auerbach RP. Improving suicide prevention through evidence-based strategies: a systematic review. Am J Psychiatry. 2021;178(7):611–24. doi: 10.1176/appi.ajp.2020.20060864 33596680 PMC9092896

[60] Wu H, Lu L, Qian Y, Jin X-H, Yu H-R, Du L, et al. The significance of cognitive-behavioral therapy on suicide: an umbrella review. J Affect Disord. 2022;317:142–8. doi: 10.1016/j.jad.2022.08.067 36041581

[61] Maddox BB, Crabbe S, Beidas RS, Brookman-Frazee L, Cannuscio CC, Miller JS, et al. “I wouldn’t know where to start”: perspectives from clinicians, agency leaders, and autistic adults on improving community mental health services for autistic adults. Autism. 2020;24(4):919–30. doi: 10.1177/1362361319882227 31674198 PMC7192780

[62] Roudbarani F, Tablon Modica P, Maddox BB, Bohr Y, Weiss JA. Clinician factors related to the delivery of psychotherapy for autistic youth and youth with attention-deficit hyperactivity disorder. Autism. 2023;27(2):415–27. doi: 10.1177/13623613221106400 35786029

[63] Lipinski S, Blanke ES, Suenkel U, Dziobek I. Outpatient psychotherapy for adults with high-functioning autism spectrum condition: utilization, treatment satisfaction, and preferred modifications. J Autism Dev Disord. 2019;49(3):1154–68. doi: 10.1007/s10803-018-3797-1 30415320

[64] Schubart JR, Camacho F, Leslie D. Psychotropic medication trends among children and adolescents with autism spectrum disorder in the Medicaid program. Autism. 2014;18(6):631–7. doi: 10.1177/1362361313497537 24165274

[65] Vohra R, Madhavan S, Sambamoorthi U. PMH69 - High prescription drug use and polypharmacy rates among adults with autism. Value Health. 2016;19(3):A194.

[66] Jobski K, Höfer J, Hoffmann F, Bachmann C. Use of psychotropic drugs in patients with autism spectrum disorders: a systematic review. Acta Psychiatr Scand. 2017;135(1):8–28. doi: 10.1111/acps.12644 27624381

[67] Knutsen J, Wolfe A, Burke BL Jr, Hepburn S, Lindgren S, Coury D. A systematic review of telemedicine in autism spectrum disorders. Rev J Autism Dev Disord. 2016;3(4):330–44. doi: 10.1007/s40489-016-0086-9

[68] Platt S, Niederkrotenthaler T. Suicide prevention programs. Crisis. 2020;41(Suppl 1):S99–124. doi: 10.1027/0227-5910/a000671 32208762

[69] Schölmerich VLN, Kawachi I. Translating the socio-ecological perspective into multilevel interventions: gaps between theory and practice. Health Educ Behav. 2016;43(1):17–20. doi: 10.1177/1090198115605309 26747715

[70] World Health Organization. Public health action for the prevention of suicide: a framework. Switzerland: World Health Organization; 2012.

[71] Robinson J. Why autistic people like me need to help shape autism science. STAT. 2017.

[72] Cramer RJ, Kapusta ND. A social-ecological framework of theory, assessment, and prevention of suicide. Front Psychol. 2017;8:1756. doi: 10.3389/fpsyg.2017.01756 29062296 PMC5640776

[73] Kirby AV, Morgan L, Hilton C. Autism and mental health: the role of occupational therapy. Am J Occup Ther. 2023;77(2). 10.5014/ajot.2023.050303 PMC10162488 36996455

[74] Wiens J, Saria S, Sendak M, Ghassemi M, Liu VX, Doshi-Velez F, et al. Do no harm: a roadmap for responsible machine learning for health care. Nat Med. 2019;25(9):1337–40. doi: 10.1038/s41591-019-0548-6 31427808

[75] Hedegaard H, Schoenbaum M, Claassen C, Crosby A, Holland K, Proescholdbell S. Issues in developing a surveillance case definition for nonfatal suicide attempt and intentional self-harm using International Classification of Diseases, Tenth Revision, Clinical Modification (ICD-10-CM) coded data. Natl Health Stat Rep. 2018;2018(108):1–19. 29616901

[76] Centers for Disease Control and Prevention, Agency for Toxic Substances and Disease Registry, Geospatial Research Analysis and Services Program. CDC/ATSDR social vulnerability index 2020, 2018, and 2016 database. In: Centers for Disease Control and Prevention AfTSaDR, Geospatial Research, Analysis, and Services Program, editor.

[77] Cutter S, Boruff B, Shirley W. Social vulnerability to environmental hazards. Soc Sci Q. 2003;84(2):242–61.

[78] Douglas MD, Bent Weber S, Bass C, Li C, Gaglioti AH, Benevides T, et al. Creation of a longitudinal legal data set to support legal epidemiology studies of mental health insurance legislation. Psychiatr Serv. 2022;73(3):265–70. doi: 10.1176/appi.ps.202100019 34320828

[79] Hand BN, Benevides TW, Carretta HJ. Suicidal ideation and self-inflicted injury in Medicare enrolled autistic adults with and without co-occurring intellectual disability. J Autism Dev Disord. 2020;50(10):3489–95. doi: 10.1007/s10803-019-04345-x 31858322

[80] Casanova MF, Frye RE, Gillberg C, Casanova EL. Editorial: comorbidity and autism spectrum disorder. Front Psychiatry. 2020;11:617395. doi: 10.3389/fpsyt.2020.617395 33329163 PMC7714785

[81] Denny JC, Bastarache L, Ritchie MD, Carroll RJ, Zink R, Mosley JD, et al. Systematic comparison of phenome-wide association study of electronic medical record data and genome-wide association study data. Nat Biotechnol. 2013;31(12):1102–10. doi: 10.1038/nbt.2749 24270849 PMC3969265

[82] Allen H, Gordon SH, Lee D, Bhanja A, Sommers BD. Comparison of utilization, costs, and quality of Medicaid vs subsidized private health insurance for low-income adults. JAMA Netw Open. 2021;4(1):e2032669. doi: 10.1001/jamanetworkopen.2020.32669 33399859 PMC9377505

[83] Rubenstein E, Bishop L. Is the autism boom headed for Medicaid? Patterns in the enrollment of autistic adults in Wisconsin Medicaid, 2008-2018. Autism Res. 2019;12(10):1541–50. 31317639 10.1002/aur.2173 PMC7006836

[84] Kirby AV, Bakian AV, Bilder DA, Keeshin BR, Coon H, editors. Diagnostic associations with suicide death: a U.S. statewide analysis of mental and physical health conditions among autistic people who died by suicide. Stockholm, Sweden: International Society for Autism Research; 2023 May 3–6.

[85] Roux AM, Chvasta K, Koffer Miller KH, Cooper D, Tao S, Assing-Murray E, et al. National Autism indicators report: introduction to Medicaid and Autism. Philadelphia (PA): A.J. Drexel Autism Institute, Drexel University; 2023 Mar.

[86] Jacobs MM, Johnson LA. Chronic disease and minimum wage: disparities in diagnosis among Black and Hispanic workers. Divers Eq Health Care. 2024;21(1).

[87] Calinski T, Harabasz J. A dendrite method for cluster analysis. Commun Stats - Theory Methods. 1974;3(1):1–27. doi: 10.1080/03610927408827101

[88] Schwarz G. Estimating the dimension of a model. Ann Stat. 1978;6(2):461–4.

[89] Husson F, Josse J, Le S, Mazet J. Package ‘FactoMineR’: multivariate exploratory data analysis and data mining; 2023 Mar 27.

[90] Benevides TW, Carretta HJ, Graves KY. Case identification and characterization of autistic young adults in 2010 Medicare fee-for-service claims. Autism Adulthood. 2019;1(3):210–8. doi: 10.1089/aut.2018.0036 36601414 PMC8992825

[91] Goldberg DM, Lin H-C. Effects of the mental health parity and addictions equality act on depression treatment choice in primary care facilities. Int J Psychiatry Med. 2017;52(1):34–47. doi: 10.1177/0091217417703289 28486877

[92] Mulvaney-Day N, Gibbons B, Alikhan S, Karakus M. Mental health parity and addiction equity act and the use of outpatient behavioral health services in the United States, 2005-2016. Am J Public Health. 2019;109(S3):S190–6. 31242013 10.2105/AJPH.2019.305023 PMC6595520

[93] Stuart EA, McGinty EE, Kalb L, Huskamp HA, Busch SH, Gibson TB, et al. Increased service use among children with autism spectrum disorder associated with mental health parity law. Health Aff (Millwood). 2017;36(2):337–45. doi: 10.1377/hlthaff.2016.0824 28167724 PMC8320748

[94] Candon MK, Barry CL, Epstein AJ, Marcus SC, Kennedy-Hendricks A, Xie M, et al. The differential effects of insurance mandates on health care spending for children’s autism spectrum disorder. Med Care. 2018;56(3):228–32. doi: 10.1097/MLR.0000000000000863 29287035 PMC5811382

[95] McMahon M, Hatton C, Bowring DL. Polypharmacy and psychotropic polypharmacy in adults with intellectual disability: a cross-sectional total population study. J Intellect Disabil Res. 2020;64(11):834–51. doi: 10.1111/jir.12775 32902029

[96] Lunsky Y, Modi M. Predictors of psychotropic polypharmacy among outpatients with psychiatric disorders and intellectual disability. Psychiatr Serv. 2018;69(2):242–6. doi: 10.1176/appi.ps.201700032 29089006

[97] Espadas C, Ballester P, Londoño AC, Almenara S, Aguilar V, Belda C, et al. Multimorbidity and psychotropic polypharmacy among participants with autism spectrum disorder with intellectual disability. Psychiatry Res. 2020;292:113321. doi: 10.1016/j.psychres.2020.113321 32738553

[98] Thalmayer AG, Friedman SA, Azocar F, Harwood JM, Ettner SL. The Mental Health Parity and Addiction Equity Act (MHPAEA) evaluation study: impact on quantitative treatment limits. Psychiatr Serv. 2017;68(5):435–42. 27974003 10.1176/appi.ps.201600110 PMC5411313

[99] Zipunnikov V, Caffo B, Yousem D, Davatzikos C, Schwartz B, Crainiceanu C. Multilevel functional principal component analysis for high-dimensional data. J Comput Graph Stat. 2011;20(4):852–73. 25960627 10.1198/jcgs.2011.10122 PMC4425352

[100] Cooper K, Smith LGE, Russell AJ. Gender identity in autism: sex differences in social affiliation with gender groups. J Autism Dev Disord. 2018;48(12):3995–4006. doi: 10.1007/s10803-018-3590-1 29705922 PMC6223803

[101] Lai J, Rhee E, Nicholas D. Suicidality in autism spectrum disorder: a commentary. Adv Neurodev Disord. 2017;1(3):190–5.

[102] Jacobs M, Briley P, Ellis C. Quantifying experiences with telepractice for aphasia therapy: a text mining analysis of client response data. Semin Speech Lang. 2020;41(5):414–32. doi: 10.1055/s-0040-1716887 32998165

[103] Ellis C, Jacobs M. The cost of social distancing for persons with aphasia during COVID-19: a need for social connectedness. J Patient Exp. 2021;8:23743735211008311. doi: 10.1177/23743735211008311 34179438 PMC8205350

[104] Marlow NM, Simpson KN, Vaughn IA, Jo A, Zoller JS, Short EB. Healthcare costs and medication adherence among patients with fibromyalgia: combination Medication vs. Duloxetine, Milnacipran, Venlafaxine, and Pregabalin initiators. Pain Pract. 2018;18(2):154–69. doi: 10.1111/papr.12585 28419725 PMC5647203

[105] Desai R, Jo A, Marlow NM. Risk for medication nonadherence among medicaid enrollees with fibromyalgia: development of a validated risk prediction tool. Pain Pract. 2019;19(3):295–302. doi: 10.1111/papr.12743 30369018

[106] Xie Z, Tanner R, Striley CL, Marlow NM. Association of functional disability with mental health services use and perceived unmet needs for mental health care among adults with serious mental illness. J Affect Disord. 2022;299:449–55. doi: 10.1016/j.jad.2021.12.040 34942217

[107] Xie Z, Tanner R, Striley CL, Marlow NM. Association of functional disability and treatment modalities with perceived effectiveness of treatment among adults with depression: a cross-sectional study. Disabil Health J. 2022;15(2):101264. doi: 10.1016/j.dhjo.2021.101264 35058170

[108] Marlow N, Xie Z, Tanner R, Jacobs M, Hogan M, Joiner T, et al. Association between functional disability type and suicide-related outcomes among U.S. adults with disabilities in the National Survey on Drug Use and Health, 2015-2019. J Psychiatr Res. 2022;153:213–22. 35841817 10.1016/j.jpsychires.2022.07.014 PMC9811968

[109] Marlow NM, Xie Z, Tanner R, Jo A, Kirby AV. Association between disability and suicide-related outcomes among U.S. adults. Am J Prev Med. 2021. 10.1016/j.amepre.2021.05.035 34465506

[110] Kramer J, Schwartz A, Hallock T, Myrvold R, Hwang I, Pfeiffer B, et al. Developing and evaluating a toolkit of strategies to support remote inclusive research teams. J Intellect Disabil. 2022. doi: 10.1177/17446295221110390 35762113

[111] McPherson G, Thorne S. Exploiting exceptions to enhance interpretive qualitative health research: insights from a study of cancer communication. Int J Qual Methods. 2006;5(2):73–86. doi: 10.1177/160940690600500210

